# Medical Protection and Defence

**Published:** 1893-12-02

**Authors:** 


### Medical Protection and Defence.

Mr. Victor Horsley, the President of the Medical Defence Union (Limited) has sent us the following statement which has been ordered by the Council to be published:—

Resumé of Negotiations re Amalgamation of the London and Counties Medical Protection Society (Limited) with the Medical Defence Union (Limited).

Informal negotiations were opened on the above matter by two letters addressed to Mr. Victor Horsley, one from Mr. G. B. Mead, one of the secretaries to the London and Counties Medical Protection Society, dated June 6th, 1893, and the other dated June 19th, 1893, from Dr. Hugh Woods, the other secretary of that society.

The first public step was a letter from Dr. Hugh Woods asking whether the Council of the Medical Defence Union would receive a deputation from the Council of the London and Counties Protection Society with a view to commencing negotiations *re* amalgamation.

This letter was considered by the Council of the Medical Defence Union on July 12th, 1893, when it was resolved, "That this letter be received and entered upon the minutes, and that Dr. Woods be informed that the Council of the Medical Defence Union will be glad to receive the Sub-Committee of the London and Counties Medical Protection Society (Limited) at their next meeting on the 26th instant."

On July 26th, 1893, a deputation from the London and Counties Protection Society, headed by the president of that society, Mr. Jonathan Hutchinson, came as delegates to lay before the Medical Defence Union the following proposals for a basis of negotiations: (*a*) That both societies should be wound up with the object of forming a third society. (*b*) That certain administrative powers should be given to the branches, but with some guarantee of economy. (*c*) That no medical official should be paid, and that, previously to the final agreement, an acturial examination of the liabilities and financial condition of each society should be made.

In the discussion that followed, the feeling was unanimously expressed on both sides, that the present system, whereby the profession was taxed with the cost of two executives for the performance of the same work, was most undesirable, and that fusion therefore should be brought about if possible.

It was pointed out, at the same time by the Council of the Medical Defence Union, that it was clearly very impolitic and contrary to the best interests of the profession that both societies should wind up, and thus for a time not only deprive medical practitioners of means of defence, but also endanger the stability of schemes of medical defence in general.

The following resolution was, after the withdrawal of the deputation, passed by the Council of the Medical Defence Union: "That a committee, consisting of the three following gentlemen, namely, Mr. Victor Horsley, Dr. Masters, and one of the secretaries, be appointed to meet a committee of the London and Counties Medical Protection Society to discuss the suggestions laid before this Council at this meeting by the deputation of the London and Counties Protection Society, this committee to have power to consult the solicitors and to present a report to this Council."

The Joint Committee thus constituted met on August 8th, 1893, after which certain informal suggestions for consideration were made by Dr. Leslie Phillips, one of the secretaries of the Medical Defence Union.

In answer to this, a letter was received by Dr. Leslie Phillips, dated August 16th, 1893, from the committee of the London and Counties Society, and signed by Dr. Woods. In this reply certain other suggestions were also made.

Following upon these negotiations the Joint Committee held a series of meetings, at one of which two solicitors were present, representing respectively the two societies.

At these meetings the following general conclusions were agreed to, and were accepted by the Council of the Medical Defence Union at their next (fortnightly) meeting:—

1. That in view of the much smaller membership and less established position of the London and Counties Protection Society it alone should wind up.

This course coincided with the opinion of the lawyers, and was, in fact, adopted under their guidance, as they showed that it is legally impossible for one society to hand over its members *en bloc*.

2. That the London and Counties Protection Society having wound up, its former members should be admitted into the Medical Defence Union in the usual way.

3. That the former Council of the London and Counties Protection Society should be liberally represented upon the Council of the Medical Defence Union. (Note.—The consideration of this matter is further detailed below.)

4. That the rights and privileges of the members of the London and Counties Protection Society were completely safeguarded by the fact that they were identical with those of the members of the Medical Defence Union.

This view was arrived at after a thorough and critical examination of the constitutions and memoranda of association of the two societies, made by the lawyers advising the Joint Committee.

5. That the London and Counties Protection Society could not legally guarantee the transference of any of its members.

6. That the accounts and liabilities of the two societies should be investigated and reported on by chartered accountants.

7. That the duties of district councils of committees should be advisory, organising, and ethical.

From the foregoing it will be seen that substantial progress was made towards an understanding on this subject, the delegates of the Medical Defence Union having at all stages referred important points at issue to their Council for advice and instruction. However, concerning the constitution of the Council of the Union after the amalgamation should have been completed, several proposals were put forward. The first of these was suggested by the delegates of the London and Counties Protection Society, who proposed that for the first eight* months after fusion the Council of the Union should consist of twelve persons nominated by the Medical Defence Union, and twelve persons nominated by the London and Counties Protection Society. These twenty-four persons were to elect from among their number a president, treasurer, secretaries, &c., for the eight months in question. At the end of the year, *e.g.*, 1894, the government of the Union was to revert to the Council of the Medical Defence Union as at present constituted.

This proposal, which, of course, necessitated the temporary destruction of the administrative machinery of the Medical Defence Union, was communicated by their delegates to the council of that society.

The Council of the Medical Defence Union felt that this proposal was unworkable for several reasons.

Firstly, that in addition to the difficulty of constituting an executive to work for such a short period out of a body of men unaccustomed to act together, the proposal would involve the complete alteration of the constitution of the union at a general meeting.

Secondly, the Council of the Medical Defence Union considered that with regard to the number of representatives of the two societies on the Council, since the rights and privileges of the members of the two societies were identical, it would be fair and equitable if the representation of each company upon the conjoint Council should be calculated according to the number of members composing each society. Since the numerical strength of the Medical Defence Union is about 2,800, and that its Council numbers 42, and whereas the numerical strength of the London and Counties Protection Society is but between 600 and 700, proportionate representation would, under ordinary circumstances, be met by the addition to the Council of the Defence Union of ten members nominated by the London and Counties Protection Society.

In view, however, of the fact that the London and Counties Society contemplated winding up, the Council of the Defence Union agreed to receive twenty nominees of the London and Counties Society.

Since the London and Counties Medical Protection Society was organised partly on the understanding that the District Councils should have wide powers, and since one or two members of the Council of the London and Counties Protection Society had made this point a *sine quâ non* in their Scheme of Medical Defence, it was clear that the fundamental difference of opinion which would of necessity arise on such an important point might be fatal to the real success of the amalgamation by impairing the efficiency and hampering the work of the conjoint Council.

The Council of the Medical Defence Union resolved therefore to ask that the list of nominees of the London and Counties Protection Society should be furnished to them before further progress was made with the negotiations.

This request however was refused by the London and Counties Protection Society, in consequence of which the negotiations were suspended.

* The number eight is taken because, whereas the financial year of the Defence Union ceases on December 31st, that of the London and Counties Society is closed on April 31st.

---

By Hugh Woods, M.D., Honorary Secretary of the London and Counties Medical Protection Society.

I have just received a document purporting to be a *résumé* of the negotiations between the Committees on Amalgamation of the above Societies, and a note from the President of the Medical Defence Union informing me that negotiations are at an end. In case this document should be sent for publication before it has been laid before the Committee of our Society, I send you an interim reply until there is time for our Council or Committee to reply formally. The Medical Defence Union has from the first disclaimed taking the initiative in promoting amalgamation, and we are proud to plead guilty to an attempt to bring about a fusion which all alike professed, and ought to desire. The statement that our Committee brought about the termination of the Conference by a refusal to accept a proposal, which could hardly have been made seriously—namely, that the names of our representatives in the suggested amalgamated society should first be submitted for approval to the Council of the Medical Defence Union is not correct. The real fact is that this proposal was met by the natural counter-proposal that the names of those proposed for the Amalgamated Council by the Medical Defence Union should in like manner be submitted to the London and Country Medical Protection Society. Is it this counter-proposal of ours that has put an end to the conference? Surely, what is sauce for the goose is sauce for the gander. Again, the statement that our Society "contemplated winding up," is an expression which used *à la* Medical Defence Union, means that our Committee considered that our Council might be asked to submit to even this self-effacement for the general good. But, under the actual circumstances, to say: "In view, however, of the fact that the London and Counties Society contemplated winding up, the Council of the Defence Union agreed, &c.," is to make a representation which is not a true one. Our Society does not contemplate winding up, and the question never has, and probably never will be, broached before our Council. On the question of the relative numbers of the two societies, I dispute the accuracy of the published figures of the Medical Defence Union, and I affirm that the present number of *bonâ fide* members of our Society is not less than 890. I may add that it has become more and more evident, at any rate to some of the members of our Council as negotiations have progressed, that amalgamation is at present undesirable, and that for several reasons. *Inter alia*, the Medical Defence Union seems disposed to act simply as a defender of individual members, whereas the Medical Protection Society desires to protect and forward the interests of the profession at large by the suppression of quacks and the like. Moreover, the Defence Union appears to be still of the mind that an oligarchy is better that a free government, and to desire to exercise all power from one centre, leaving its branches helpless, except in so far as they may be paying supporters of the central authority, and still further differs from the London and Counties Society in the opinion that all the medical officers should be unpaid, as insisted on by our Society. These and other differences which emerged during the negotiations appeared to render undesirable the amalgamation of the two societies. If the Medical Defence Union is resolved to retain unmodified its absolute central authority, its limited range of operation, and its paid medical officials, so is the London and Counties Medical Protection Society determined to stand by its more representative principles, its wider activities, and its honorary administration

